# Allergic Contact Dermatitis From Transient Formaldehyde Exposure in a Traveler: Are All Backpacks Created Equal?

**DOI:** 10.7759/cureus.12252

**Published:** 2020-12-24

**Authors:** Flavia Valdes, Stephanie McNamara, Jonette Keri

**Affiliations:** 1 Dermatology, University of Miami Miller School of Medicine, Miami, USA; 2 Dermatology, University of Miami Health System, Miami, USA

**Keywords:** contact dermatitis, formaldehyde, hypersensitivity reaction, backpack

## Abstract

Contact dermatitis is an increasingly common dermatologic condition, and it is generally associated with exposure to an irritant or allergen that causes pruritic skin lesions of varying size and severity. Formaldehyde, frequently found in household products, preservatives, and fabrics, is a common trigger for allergic contact dermatitis (ACD). We report the unique case of an ACD flare in a patient, which was caused by contact with a backpack while he was traveling abroad. The patient’s right arm was in contact with the backpack’s shoulder straps every time he would put on the backpack. As a consequence, he developed a pruritic erythematous rash on his right arm. The patient had undergone patch testing prior to the trip, which had shown a positive reaction to formaldehyde, among other compounds. The patient started noticing the resolution of the rash once he stopped using this specific backpack.

## Introduction

Contact dermatitis is a skin reaction characterized by pruritic skin lesions ranging from transient erythema to vesicles and bullae in areas of contact with a foreign substance [1]. It can be classified into two different categories: irritant and allergic. Although both are very similar in terms of their clinical presentation, they differ in that irritant contact dermatitis (ICD) does not require prior exposure to sensitization. ICD occurs when there is direct damage to the keratinocytes, which induces the release of inflammatory cytokines and the development of an eczematous rash. Allergic contact dermatitis (ACD), on the other hand, occurs primarily as a result of a type-IV, T-cell mediated, delayed hypersensitivity reaction [2]. Common substances that may elicit this type of response in ACD include formaldehyde, poison ivy, and nickel. The most important step in the management of contact dermatitis is the identification and avoidance of the trigger substance(s). Topical steroids are helpful in the treatment of localized reactions but should be avoided in chronic episodes or those that continue to recur. Topical immunomodulators, ultraviolet (UV) therapy, and retinoids are some of the other pharmacological options available for the treatment of contact dermatitis [3].

## Case presentation

In May of 2019, a 45-year-old male presented to our dermatology clinic for evaluation and management of his contact dermatitis and rosacea after a recent hospitalization in April. The patient had developed a facial contact dermatitis impetiginized with Tzanck-positive herpes zoster and a rosacea flare. The patient had been using valaciclovir, triamcinolone ointment, and petroleum jelly for one week after hospitalization, which had cleared the facial redness. However, a month after hospitalization, the patient had noticed worsening facial redness as well as itching on the body more pronounced than on the face. He had been subsequently diagnosed with scabies by his primary care physician and successfully treated. On physical exam, the patient had pruritic smooth red plaques on the cheeks and glabella. There were red papules on the nose and small pink papules on the trunk, upper extremities, and posterior neck. He was employed as an operations manager in construction and reported working indoors only of late. After the initial consult, the patient was started on ivermectin 1% cream daily and doxycycline 100 mg twice a day for the treatment of rosacea. Patch testing was also recommended by his dermatologist after the patient stated that he had been applying paper towels to his face about 100 times a day throughout the years, and once he had replaced this with the use of gauze, he felt that his facial redness had improved. Results of the patch test showed a positive reaction to clobetasol-17-propionate, cocamidopropyl betaine, decyl glucoside, formaldehyde, and methoxycinnamates. A list of safe products was provided to the patient in order to better manage and control his ACD and there was a significant improvement in his condition with the avoidance of the trigger agents.

**Figure 1:**
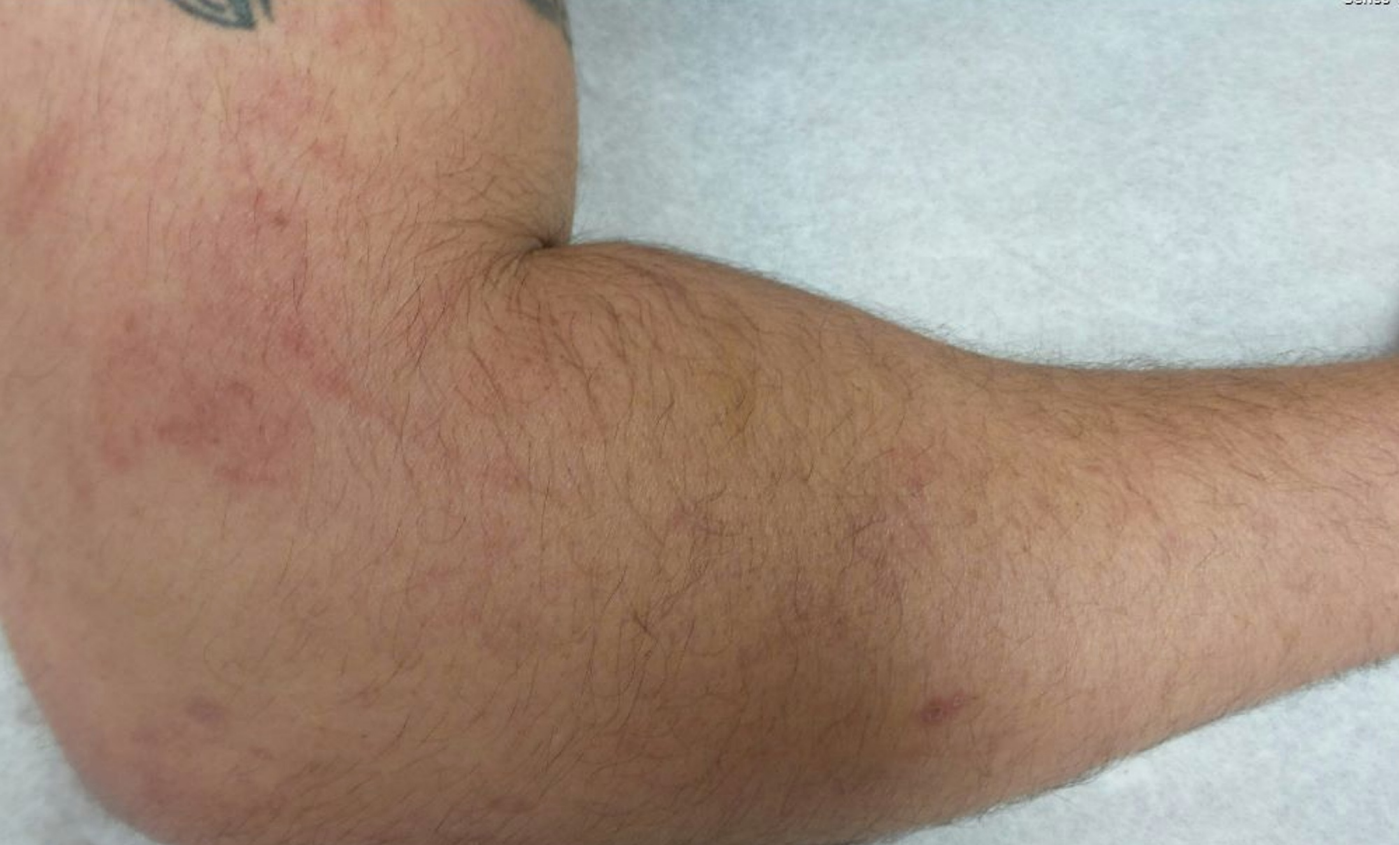
Patient’s right arm with allergic contact dermatitis induced by the contact with shoulder straps on his travel backpack

At a follow-up appointment seven months later, the patient had recently returned from a trip to Europe. On day three of the trip, he had noticed a localized pruritic erythematous maculopapular rash with well-demarcated borders on his right arm (Figure 1), which he had later recognized as being triggered by the backpack he had been using at that time. The patient had been able to make this connection because he had realized that every time he had put his backpack on, one of the shoulder straps had made contact with the arm that had developed the skin lesions (right) but not with the left arm, which had consequently not been affected. His contact dermatitis had begun to resolve with the avoidance of the use of this specific backpack.

## Discussion

Formaldehyde resins have been widely used in the textile industry as an anti-wrinkle and crease-resistant component since the 1920s [4]. ACD caused by formaldehyde exposure has become increasingly prevalent over the years, and there have been several published reports describing the use of formaldehyde in the textiles industry as well as its cutaneous adverse effects. ACD may develop as a response to formaldehyde exposure. It usually presents as a pruritic erythematous skin eruption as seen in our patient. The arms, legs, and posterior thighs are some of the most commonly affected areas. The amount of formaldehyde found in most products is extremely small; however, formaldehyde is also a natural product of human metabolism, and trace amounts of this chemical can be found in the environment, construction materials, and in our bodies [5]. Given the patient’s positive patch test reaction to formaldehyde, as well as the known use of this chemical in many fabrics, it is possible that one of the components of the backpack the patient had been using while on vacation had caused him to have a flare of his contact dermatitis after repeated exposure to the offending agent.

A study published in 2020 collected results from 2,373 patients who underwent patch testing between 1990 and 2016 at the Massachusetts General Hospital. Results from patients who underwent the testing between January 2007 and December 2016 were compared with data collected between 1998-2006 and 1990-2006 as well as with data reported by the North American Contact Dermatitis Group with the goal to assess the different trends in patch testing results. The top allergen identified for all time periods was nickel, with 19.8% of the subjects testing positive for it between 2007-2016. Only 2.5% of the patients in this cohort tested positive for formaldehyde, while 5.8% of patients tested between 1998-2006 had a positive patch test for formaldehyde, indicating a decrease in sensitization rate for formaldehyde over time [6]. A cross-sectional study in Denmark assessing 10-year trends for contact dermatitis related to formaldehyde examined patch test results from 8,463 patients and determined the prevalence of contact dermatitis to 1% formaldehyde, which was 1.5%, which also demonstrates a decreasing trend in formaldehyde-induced contact dermatitis over this time period. No trend was observed with 2% formaldehyde [7]. This shows that contact dermatitis caused by formaldehyde is not common in the United States as well as other countries and that there has been an overall decrease in sensitization rates to this compound.

While there are no regulations in the United States limiting the levels of formaldehyde in clothing items, there has been a documented decline, which can be attributed to the use of low-formaldehyde resins in manufacturing companies as a result of the rising awareness regarding the various health risks associated with formaldehyde such as its carcinogenic properties [8]. It is important to note that a number of patients with positive patch test reactions to formaldehyde solutions fail to develop a reaction to textiles containing formaldehyde. This could be attributed to a degree of sensitivity low enough to not allow for the development of contact dermatitis upon being exposed to formaldehyde-containing fabrics [4].

## Conclusions

ACD is a type-IV hypersensitivity reaction commonly elicited by substances such as formaldehyde, poison ivy, and nickel. Although formaldehyde is used in small amounts in the textiles industry as a preservative, it is important for patients diagnosed with ACD to be aware of this connection to prevent recurrence despite the fact that not everyone with a positive formaldehyde patch test has a reaction to formaldehyde in textiles. Management of this condition should focus on avoiding the trigger substances and the use of topical steroids for localized diseases. In this report, we presented a unique case of an episode of ACD triggered by the use of a travel backpack. We reviewed the patient's known allergens and considered possible exposures to the same from the backpack. From this, we concluded that the most likely culprit behind the patient's ACD was formaldehyde. This case report serves as a reminder to consider formaldehyde in skin eruptions in cases where the patient has had exposure to backpacks or other similar types of personal items.
